# Supplementary material for: Polyketide synthase gene clusters in the ericoid mycorrhizal fungus Oidiodendron maius: genome-wide prediction, expression profiling, and functional characterization of knockout mutants
Source: Front Plant Sci. 2026 Sep 16;17:1924430. doi: 10.3389/fpls.2026.1924430 (PMC13623903; doi:10.3389/fpls.2026.1924430)
Supplement: Supplementary file 1 [file SupplementaryFile1.zip › Supplementary Materials/Supplementary Folder 1.zip!/Oidma1_AssemblyScaffolds_Repeatmasked/knownclusterblast/cluster17/jgi.p_Oidma1_53060_mibig_hits.html]

| MIBiG Protein | Description | MIBiG Cluster | MiBiG Product | % ID | % Coverage | BLAST Score | E-value |
| --- | --- | --- | --- | --- | --- | --- | --- |
| AAG27132.1 | Fum6p | BGC0000062 | Fumonisin\_biosynthetic\_gene\_cluster | 41 | 103.355079217 | 847 | 1.2e-245 |
| ACB12553.1 | Fum6 | BGC0000063 | Fumonisin\_biosynthetic\_gene\_cluster | 40 | 103.261882572 | 834 | 1.1e-241 |
| AAM54108.1 | cytochrome\_P450 | BGC0000020 | Ansamitocin\_biosynthetic\_gene\_cluster | 38 | 100.279589935 | 638 | 1.9e-182 |
| ACY13429.1 | cytochrome\_P450 | BGC0001367 | Haliamide\_biosynthetic\_gene\_cluster | 28 | 40.2609506058 | 141 | 7.4e-33 |
| BAI44339.1 | P450 | BGC0000677 | Cyclooctatin\_biosynthetic\_gene\_cluster | 32 | 21.9944082013 | 131 | 4.5e-30 |
| CAQ18836.1 | putative\_cytochrome\_P450 | BGC0000954 | Ajudazol\_biosynthetic\_gene\_cluster | 26 | 37.5582479031 | 126 | 2.5e-28 |
| CAQ18837.1 | putative\_cytochrome\_P450 | BGC0000954 | Ajudazol\_biosynthetic\_gene\_cluster | 33 | 20.3168685927 | 116 | 2e-25 |
| AAC49958.1 | cytochrome\_P450 | BGC0001279\_c2 | Deoxysambucinol\_/\_sambucinol\_/\_roridin\_E\_biosynthetic\_gene\_cluster | 27 | 33.1780055918 | 92 | 3e-18 |
| AGO65988.1 | putative\_cytochrome\_p450 | BGC0000992 | Fusaridione\_A\_biosynthetic\_gene\_cluster | 29 | 20.1304753029 | 89 | 2e-17 |
| AAC49200.1 | putative\_p450\_monooxygenase | BGC0000152 | Sterigmatocystin\_biosynthetic\_gene\_cluster | 28 | 28.238583411 | 89 | 3.4e-17 |
| EED17999.1 | benzoate\_4-monooxygenase\_cytochrome\_P450,\_putative | BGC0000154 | Stipitatic\_acid\_biosynthetic\_gene\_cluster | 23 | 30.9412861137 | 84 | 8.3e-16 |
| AAS79446.1 | P450 | BGC0000035 | Chalcomycin\_biosynthetic\_gene\_cluster | 27 | 20.7828518173 | 66 | 3e-10 |
| AFI57027.1 | cytochrome\_p450 | BGC0000133 | Quartromicin\_biosynthetic\_gene\_cluster | 24 | 20.5964585275 | 56 | 3.1e-07 |
